# Research on the mechanism of core acupoints in electroacupuncture for functional constipation based on data mining and network acupuncture

**DOI:** 10.3389/fmed.2024.1482066

**Published:** 2024-12-11

**Authors:** Shun Seng Ong, Ting Tang, Lianjie Xu, Canwei Xu, Qi Li, Xiaoyue Deng, Peihua Shen, Yi Chen, Yang Song, Hai Lu, Ling Fang

**Affiliations:** ^1^Department of Traditional Chinese Medicine, Nanjing Drum Tower Hospital, Clinical College of Nanjing University of Chinese Medicine, Nanjing, China; ^2^Department of Chinese Medicine, The Affiliated Taizhou’s People Hospital of Nanjing Medical University, Taizhou, China

**Keywords:** electroacupuncture, Functional Constipation (FC), data mining, network acupuncture, association analysis

## Abstract

**Aim:**

Functional Constipation (FC) is a common gastrointestinal disorder that imposes a considerable strain on global health. It negatively impacts the quality of life and results in significant healthcare expenditures. Current treatments, such as lifestyle changes and medications, fail to meet patient satisfaction due to efficacy and safety issues. Electroacupuncture (EA), with its precise stimulation control and standardized protocols, shows promise in FC management. However, optimal EA parameters for FC treatment are yet to be established. Our study reviews EA applications in FC to inform a standardized treatment approach and explore EA’s therapeutic mechanisms.

**Methods:**

This comprehensive study utilized research literature from databases including PubMed, Embase, OVID, Web of Science, the Cochrane Library, CNKI, VIP, and Wanfang to perform a descriptive analysis of acupoint selection and EA parameters. It proceeded to analyze high-frequency acupoint groupings and stimulus parameters, followed by the excavation and analysis of core acupoint prescriptions. Subsequent steps integrated potential target identification for these core formulas, the assembly of a “core acupoint-prescription-target-constipation” network, and the construction of a protein–protein interaction (PPI) network to extract central targets. Additionally, Gene Ontology (GO) and KEGG enrichment analyses were conducted to prognosticate the underlying mechanisms by which EA may exert its therapeutic effects on FC.

**Results:**

In our study, we analyzed 141EA prescriptions for FC and identified a core set of acupoints including Tianshu (ST25), Fujie (SP14), Shangjuxu (ST37), and Zusanli (ST36) through data mining. The frequency of use was highest for Tianshu (ST25) with 119 occurrences, followed by Fujie (SP14) with 59, Shangjuxu (ST37) with 42, and Zusanli (ST36) with 23. PPI network analysis revealed key targets such as NFKB1, IL6, MyD88, TLR4, TNF, TLR2, and IL1B. GO and KEGG analyses of 49 constipation-associated targets identified 257 BP, 37 CC, and 41 MF terms, and 154 significant pathways, with the top 20 visualized for further analysis.

**Conclusion:**

The core acupoint prescription of EA for FC can exert its therapeutic effects by acting on multiple targets and pathways synergistically especially on NFKB1, IL6, MyD88, TLR4, TNF, TLR2, and IL1B. The research findings have preliminarily validated the fundamental effects and related mechanisms of EA parameters and core prescriptions, providing direction for further in-depth exploration of the mechanisms of action.

## 1 Introduction

Functional Constipation (FC) is a common gastrointestinal disorder affecting approximately 14% of the global population (1, 2). It is categorized under chronic constipation by the ROME IV criteria and is associated with a significant reduction in quality of life and increased risks of serious diseases such as colon cancer and cardiovascular issues (3–5). The economic burden is substantial, with annual healthcare costs exceeding $2.3 billion in the United States alone (6).

Despite various treatment options, including lifestyle adjustments and medications, many patients remain dissatisfied with the efficacy and side effects of conventional therapies (7–9). There is a clear need for alternative treatments that are both effective and safe. Electroacupuncture (EA), an innovative adaptation of traditional acupuncture, offers a potential solution (10). It combines needle insertion with electrical stimulation, providing precise control and standardized therapeutic protocols (11). EA has been shown to improve quality of life and bowel movement frequency in FC patients, with high safety and long-lasting efficacy (12, 13). However, the optimal EA parameters for treating FC remain unclear, and there is a scarcity of research dedicated to identifying these parameters (13, 14).

The lack of well-defined EA parameters poses significant clinical challenges. Firstly, it hinders the replication of studies and the generalization of findings, as the efficacy of EA may be contingent upon the specific parameters used. Secondly, the absence of standardized parameters can lead to inconsistent treatment outcomes, potentially undermining the credibility of EA as a viable treatment option for FC. Furthermore, the variability in EA parameters may also contribute to the difficulty in obtaining regulatory approval for EA as a standardized treatment, given the need for clear, evidence-based protocols.

Our study addresses this gap by reviewing the literature on EA’s application in treating FC over the past decade. Utilizing data mining techniques, we extracted and analyzed various EA parameters reported in the articles, including waveforms, current frequency, and acupoints. We employed the concept of network acupuncture to distill the core acupuncture prescriptions and their potential targets for FC from existing literature.

By constructing a sophisticated biological network that interconnects “acupoints-targets-constipation,” our study systematically dissects and anticipates the underlying mechanisms behind these acupuncture effects. The goal is to establish a framework that could guide the standardization of EA and enhance its clinical application, offering innovative approaches in the foundational research of constipation and clinical insights that could enhance treatment strategies.

## 2 Methods

### 2.1 Data mining

#### 2.1.1 Literature search strategy

The study utilized a scientific database from the Nanjing University of Chinese Medicine in China to conduct an extensive literature review. We searched across multiple databases including PubMed, Embase, OVID, Web of Science, the Cochrane Library, CNKI, VIP, and Wanfang, covering the period from 2013 to December 2023. To enhance the precision and sensitivity of our literature retrieval, we employed Medical Subject Headings (MeSH) terms from PubMed as the basis for our search strategy. The search strategy was carefully designed to include a broad spectrum of relevant terms. The keywords we used included “*electroacupuncture*,” “*electrical acupuncture*,” “*electrical stimulation*,” “*functional constipation*,” “*chronic constipation*,” “*slow transit constipation*,” “*dyssynergic defecation*,” “*constipation*,” “*colonic inertia*,” and “*dyschezia*” with adjustments made to accommodate the specific search requirements of each database. To ensure a thorough review, we performed manual searches in addition to our initial database queries to identify any potentially overlooked studies. Our search criteria were inclusive, not restricting by language, to ensure a broad and comprehensive analysis of the literature. The manual search was conducted by the first and second authors (Shun Seng Ong, Lianjie Xu), with any discrepancies resolved by the corresponding author (Hai Lu).

#### 2.1.2 Inclusion and exclusion criteria

The inclusion criteria were as follows: (1) Type of Study: Randomized controlled trials (RCTs) literature on the treatment of Functional Constipation with electroacupuncture, published domestically and internationally; (2) Subjects of Research: Participants diagnosed with FC, with no restrictions on age, gender, ethnicity, nationality, disease course, or source of medical history. The diagnostic criteria refer to the diagnostic criteria for FC in the Rome III criteria or the diagnostic criteria for FC in the Rome IV criteria; (3) Intervention and Control Measures: The intervention for the experimental group primarily involves electroacupuncture therapy or a combination of electroacupuncture with other traditional Chinese and Western medical therapies, focusing on acupoints from the 14 main meridians, excluding auricular acupuncture, wrist-ankle acupuncture, scalp acupuncture, etc.; The control group receives intervention measures other than electroacupuncture, which may include different acupuncture therapies, traditional Chinese medicine, Western medicine, placebos, blank controls, etc.

The exclusion criteria were as follows: (1) Subjects of Research: The diagnostic criteria for the subjects are self-devised and do not conform to the diagnostic criteria for FC; (2) Duplicate Publications: Only one instance of duplicate literature will be included; (3) Types of Literature: Abstracts, expert treatment experiences, basic experiments, theoretical discussions, summary literature, systematic reviews, meta-analyses, case reports, and review articles.

#### 2.1.3 Management of literature quality

Quality control was rigorously applied to the management of literature. Initially, the titles and abstracts of all identified articles were examined by two independent reviewers (LianJie Xu, Qi Li). Any articles that did not fulfill the established criteria were subsequently excluded. The second round of screening was conducted through a thorough reading of the full texts of the remaining articles. Ultimately, two researchers (Canwei Xu, Yi Chen) were tasked with the comprehensive review. Any discrepancies encountered during the process were addressed through discussions between the two reviewers, or by consulting a third reviewer (Hai Lu) if necessary.

#### 2.1.4 Data extraction

Microsoft Excel 2019 was utilized to create the database. The selection of acupoints, duration of stimulation, frequency of current, and type of wave were all documented within the database accordingly. Following the data entry process, a proofreading step was conducted to verify the accuracy of the information entered.

#### 2.1.5 Data analysis of data mining

Excel 2019 was employed for the descriptive statistics of the included literature, focusing on the selection of acupoints, the frequency of the current, the type of wave used, and the duration of stimulation. SPSS Modeler 18.0 was utilized to conduct the association analysis of the acupoints. The outcomes derived from the association rule analysis of core acupoint prescriptions will be graphically depicted through the utilization of Cytoscape 3.10.2 software. The analysis aimed to explore the high-frequency acupoint groups while also uncovering the core prescriptions.

### 2.2 Network acupuncture

#### 2.2.1 Core acupoint prescription—target database construction

Utilizing the acupoint names from the core prescriptions, combined with keywords like “experimental,” “molecular,” and “mechanism,” we conducted searches within the database described in section 1.1. This search spanned from 2013 to 2023. For each core acupoint and its related target, we created separate databases. The criteria for database inclusion were as follows:

1. Acupoint inclusion criteria: The study encompasses one or more acupoints from the core prescription exclusively, without the inclusion of additional acupoints or concomitant medication within the research group.
2. Study category: Animal-based experimental studies.
3. Disease focus of the study: Constipation related diseases.
4. Outcome: The targets of action are clearly defined, and the results show statistically significant changes.

The targets included must undergo validation via the UniProt database^1^ to ascertain standardized gene nomenclature that is both verified and human-specific.

#### 2.2.2 Identification and network construction of potential targets for core acupoint therapy in constipation

The keyword “constipation” was utilized to perform a search within the GeneCards^2^ database. Targets with a relevance index of two or less times the median were filtered out to enhance the relevance of the targets obtained. Supplementary target identification was conducted using the TTD^3^ and DisGeNET^4^ databases. The Evenn^5^ platform was employed to identify the intersection of acupoint and disease targets, thereby pinpointing the potential targets for core acupoint therapy in treating constipation. Visualization of the “core acupoint-targets-constipation” network was achieved using Cytoscape 3.10.2 software.

#### 2.2.3 Construction of protein–protein interaction (PPI) network

The STRING 11.5 database was leveraged to acquire PPI data, with the species filter set to “Homo Sapien” and the interaction score threshold defined as “high confidence.” Targets that were not interconnected were concealed. The derived PPI network was then imported into Cytoscape 3.10.2 software, where the Network Analyzer tool was utilized for the visualization and extraction of core proteins within the network.

#### 2.2.4 Gene ontology (GO) functional enrichment and KEGG pathway enrichment analysis

Bioinformatics enrichment analysis employs the DAVID database^6^ to conduct KEGG pathway enrichment analysis and Gene Ontology (GO) enrichment analysis for the pivotal targets associated with core acupoint prescriptions and diseases, encompassing biological processes (BP), cellular components (CC), and molecular functions (MF). The top 10 enrichment results with a *p*-value threshold of less than 0.01 are selected. The outcomes of the study are then visualized using Bioinformatics tools^7^ to generate bar and bubble charts for an illustrative analysis.

## 3 Results of data mining

### 3.1 Study identification and acquisition

**Figure 1:**
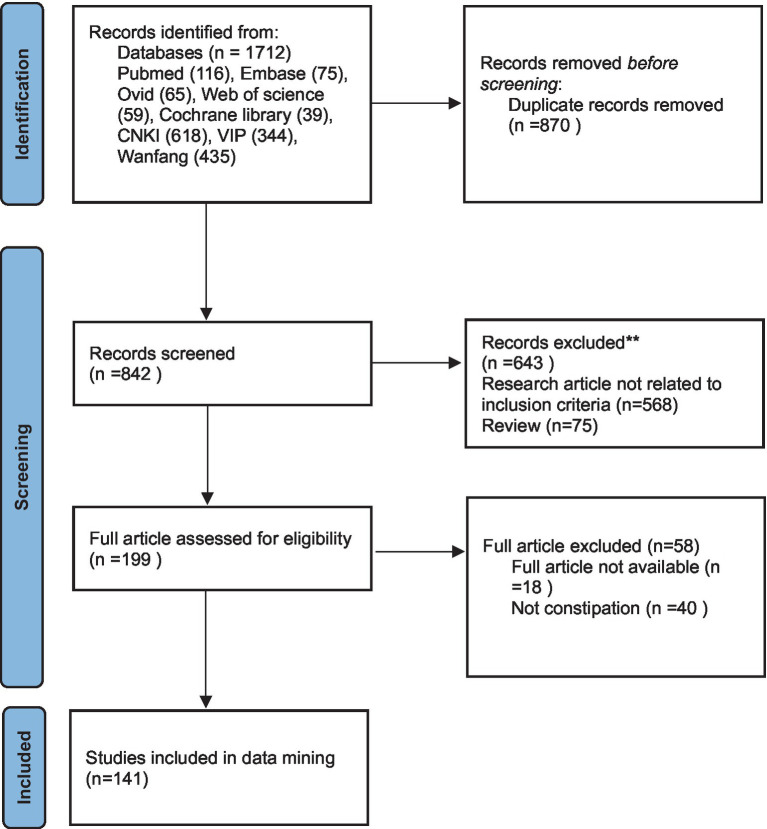
Flow diagram for selection of articles.

The initial search encompassed eight databases, identifying 1,712 pertinent articles with distributions as follows: PubMed (116), Embase (75), Ovid (65), Web of Science (59), Cochrane Library (39), CNKI (618), VIP (344), and Wanfang (435). The deduplication process reduced this number to 842 articles. Subsequent primary screening based on titles and abstracts narrowed it down to 199 articles, from which 58 were excluded after full-text evaluation. Ultimately, 141 articles met the inclusion criteria for further analysis. The exclusion criteria and the selection process are illustrated in Figure 1.

### 3.2 Frequency analysis of acupoint utilization

In this study, a total of 41 acupoints were included in the acupuncture prescriptions, with a total frequency of 381 occurrences. The most frequently used acupoints were Tianshu (ST25) with 119 times (31.23%), Fujie (SP14) with 59 times (15.48%), Shangjuxu (ST37) with 42 times (11.02%), and Zusanli (ST36) with 23 times (6.03%). Table 1 illustrates the ranking of the top ten most frequently utilized acupoints.

**Table 1:** Top 10 most frequently utilized acupoints for treating FC.

Number	Acupoints	Frequency (%)
1	Tianshu (ST25)	119 (31.23)
2	Fujie (SP14)	59 (15.48)
3	Shangjuxu (ST37)	42 (11.02)
4	Zusanli (ST36)	23 (6.03)
5	Zhigou (SJ6)	15 (3.93)
6	Dachangshu (BL25)	14 (3.67)
7	Ciliao (BL32)	9 (2.36)
8	Guanyuan (RN4)	9 (2.36)
9	Zhongliao (BL33)	9 (2.36)
10	Daheng (SP15)	8 (2.09)

### 3.3 Results of the association rule analysis

#### 3.3.1 Analysis of high-frequency acupoint combinations

In the context of big data, the analysis of association rules reveals the extent of mutual dependency or connections among various elements. By examining metrics such as confidence, support, and lift, one can uncover the patterns of interrelated usage among acupoints. The association analysis focused on high-frequency acupoints, with parameters set for a minimum support level of 5, a minimum confidence level of 80, a minimum lift of ≥1, a maximum of 2 antecedents. Utilizing the Apriori algorithm, a total of 14 association rules were identified, as detailed in Table 2. The results indicate that there is a stronger association between Tianshu (ST25) and Fujie (SP14), followed by an association between Tianshu (ST25) and Zusanli (ST36).

**Table 2:** Association rules analysis of high-frequency acupoints.

Number	Association rules	Count	Support (%)	Confidence (%)	Lift
1	Tianshu (ST25) → Fujie (SP14)	59	41.84	98.31	1.16
2	Tianshu (ST25) → Zusanli (ST36)	23	16.31	86.96	1.03
3	Tianshu (ST25) → Zusanli (ST36) + Shangjuxu (ST37)	16	11.35	93.75	1.11
4	Tianshu (ST25) → Dachangshu (BL25)	14	9.93	92.86	1.10
5	Tianshu (ST25) → Zhigou (SJ6) + Shangjuxu (ST37)	10	7.09	90.00	1.07
6	Zhongliao (BL33) → Ciliao (BL32)	9	6.38	88.89	13.93
7	Ciliao (BL32) → Zhongliao (BL33)	9	6.38	88.89	13.93
8	Tianshu (ST25) → Guanyuan (RN4)	9	6.38	88.89	1.05
9	Tianshu (ST25) → Dachangshu (BL25) + Shangjuxu (ST37)	9	6.38	100.00	1.18
10	Tianshu (ST25) → Fujie (SP14) + Shangjuxu (ST37)	9	6.38	100.00	1.18
11	Tianshu (ST25) → Daheng (SP15)	8	5.67	100.00	1.18
12	Xialiao (BL34) → Ciliao (BL32) + Zhongliao (BL33)	8	5.67	87.50	17.63
13	Shangliao (BL31) → Ciliao (BL32) + Zhongliao (BL33)	8	5.67	87.50	17.63
14	Tianshu (ST25) → Dachangshu (BL25) + Zusanli (ST36)	8	5.67	87.50	1.04

#### 3.3.2 Core acupoint discovery based on the Apriori algorithm

**Figure 2:**
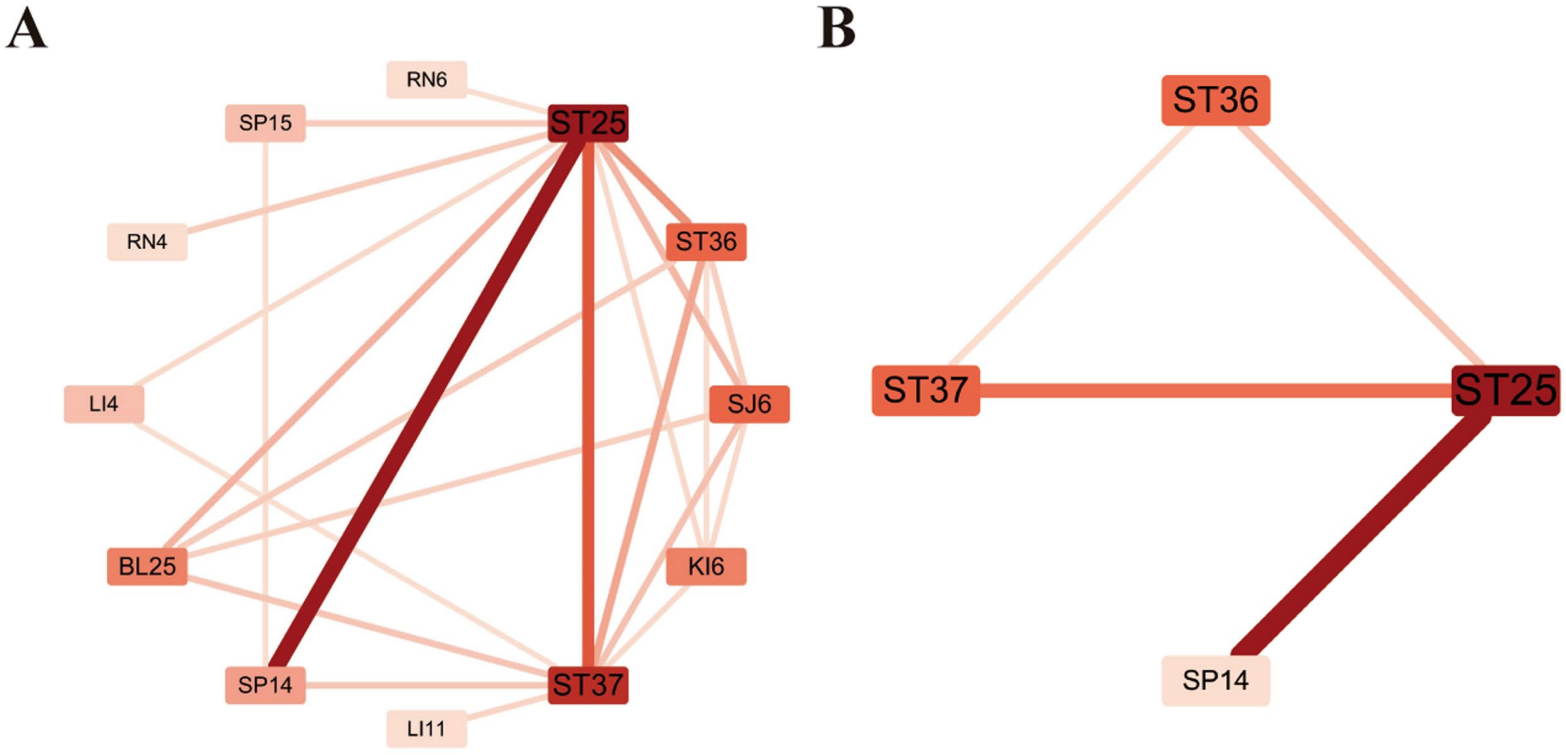
Network diagram of core acupoint prescriptions for the treatment of FC. **(A)** Connectivity > 4. **(B)** Connectivity > 15.

Utilizing “complex network” from Apriori algorithms for the analysis of core prescriptions, the core acupoints for EA treatment of FC were identified as Tianshu (ST25), Fujie (SP14), Zusanli (ST36), and Shangjuxu (ST37) as shown in Figure 2.

### 3.4 Results of EA parameter

#### 3.4.1 EA waveform

In an analysis of 141 studies, 139 explicitly described the waveforms they utilized. Dilatational waves were significantly favored, being reported in 76.97% of the studies. This usage was considerably higher than that of continuous waves, found in 20.14% of the cases, and discontinuous waves, which were mentioned in a mere 2.87% of the studies. The distribution of waveform use is summarized in Table 3.

**Table 3:** Frequency of EA waveforms.

Number	Waveform	Count	Frequency (%)
1	Dilatational wave	107	76.97
2	Continuous wave	28	20.14
3	Discontinuos wave	4	2.87

#### 3.4.2 EA current

Out of 141 studies, 111 specified the EA current used. A total of 17 different current frequencies were mentioned across these studies. The top three frequencies were 2/15 Hz, accounting for 44.14% of the studies, followed by 20 Hz at 7.2%, and 15 Hz at 6.3%. The top five frequencies of EA current are detailed in Table 4.

**Table 4:** Top 5 EA current mentioned in the studies.

Number	Frequency of EA current (Hz)	Count	Frequency (%)
1	2/15	49	44.14
2	20	8	7.2
3	15	7	6.3
4	10/50	7	6.3
5	2/50	7	6.3

#### 3.4.3 EA stimulation duration

In the 141 studies, the duration of EA stimulation during the treatment process was mentioned. There were five distinct durations reported across the studies: 30 min (90.78%), 20 min (4.96%), 25 min (2.12%), 15 min (1.41%) and 40 min (0.7%), as summarized in Table 5.

**Table 5:** EA stimulation duration mentioned in the studies.

Number	Stimulation duration (min)	Count	Frequency (%)
1	30	128	90.78
2	20	7	4.96
3	25	3	2.12
4	15	2	1.41
5	40	1	0.7

#### 3.4.4 Association analysis of EA parameters

The association analysis was conducted on three parameters—waveform, current frequency, and stimulation duration—with the following parameter settings: a minimum support level of 10, a minimum confidence level of 80, a minimum lift of 1 or higher, and a maximum of 2 antecedents. Using the Apriori algorithm, a total of 4 association rules were identified, as detailed in Table 6. The results indicate that the combination of a dilatational waveform, 2/15 Hz current frequency, and 30-min stimulation duration was most commonly applied for EA treatment of FC.

**Table 6:** Association analysis of EA parameters.

Number	Association rules	Count	Support (%)	Confidence (%)	Lift
1	30 min → Dilatational wave	84	75.68	91.67	1.02
2	Dilatational wave → 2/15 Hz	49	44.14	95.92	1.27
3	Dilatational wave → 2/15 Hz + 30 min	43	38.74	95.35	1.26
4	30 min → 20 Hz	8	7.21	100	1.11

## 4 Results of network acupuncture analysis

### 4.1 Establishment of potential target database for core acupoint prescriptions

After the data-mining and selection process, 75 literature that met the Core acupoint prescription—target database entry criteria was identified, with 58 entries for Tianshu (ST25), 37 for Shangjuxu (ST37), and 17 for Zusanli (ST36). Notably, there were no entries found for Fujie (SP14). Following collection, verification, and deduplication, the respective acupoints were linked to the following number of targets: 42 for Tianshu (ST25), 35 for Shangjuxu (ST37), and 13 for Zusanli (ST36). Fujie (SP14) was excluded from the core prescription due to the absence of any matching targets from the databases.

### 4.2 Construction of core acupoint prescription—targets- constipation network

**Figure 3:**
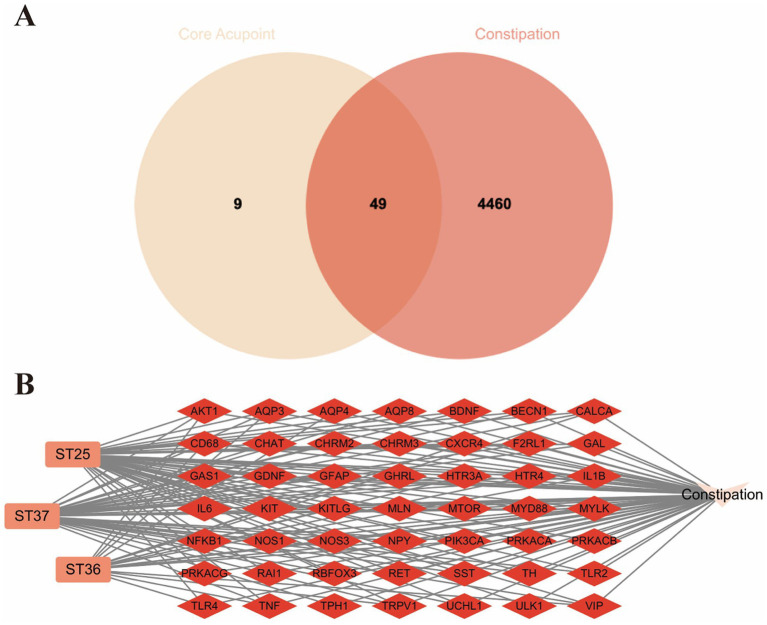
**(A)** Venn diagram of the potential target of EA treatment for FC. **(B)** Core prescription network. ST25: Tianshu; ST37: Dachangshu; ST36: Zusanli.

A search in the Genecards database yielded 6,200 targets associated with constipation, from which those scoring 2 or below in relevance were eliminated. The target list was further augmented with 10 entries from the TTD database and 425 from the Disgenet database. Post-deduplication, the final count of constipation-related targets obtained was 4,509. By intersecting with the 58 targets from the core acupoint prescription, as illustrated by Venn Diagram in Figure 3A, a final set of 49 potential targets for the acupuncture intervention in constipation was obtained. The network of core acupoint prescriptions and their associated targets in the context of constipation is depicted in Figure 3B, utilizing the Cytoscape 3.10.2 software for visualization.

### 4.3 Protein–protein interaction network analysis

**Figure 4:**
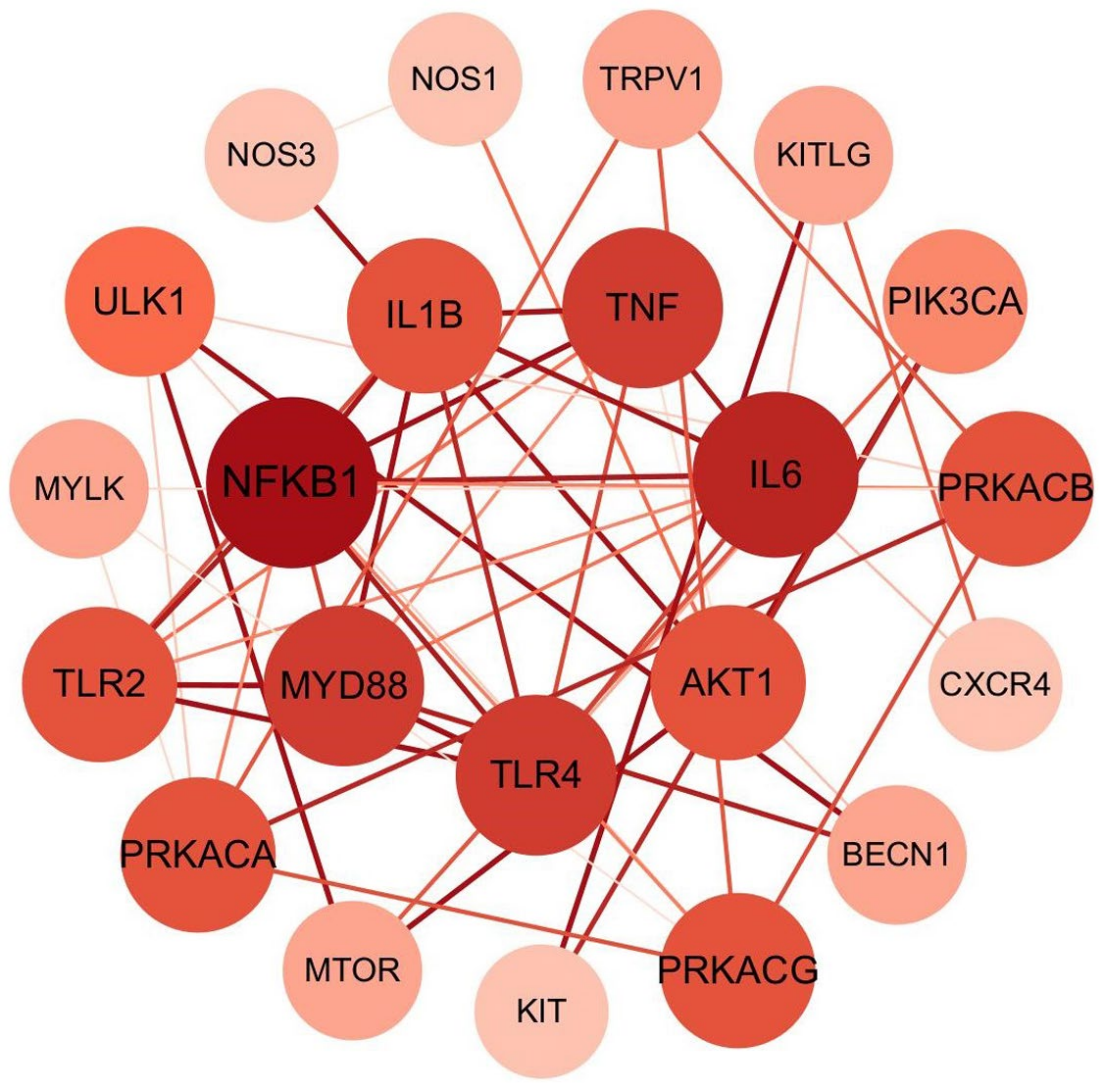
PPI network of core acupoint prescription in treating FC. The closer a node to the center, the greater its degree. PPI, Protein–protein interactions.

**Figure 5:**
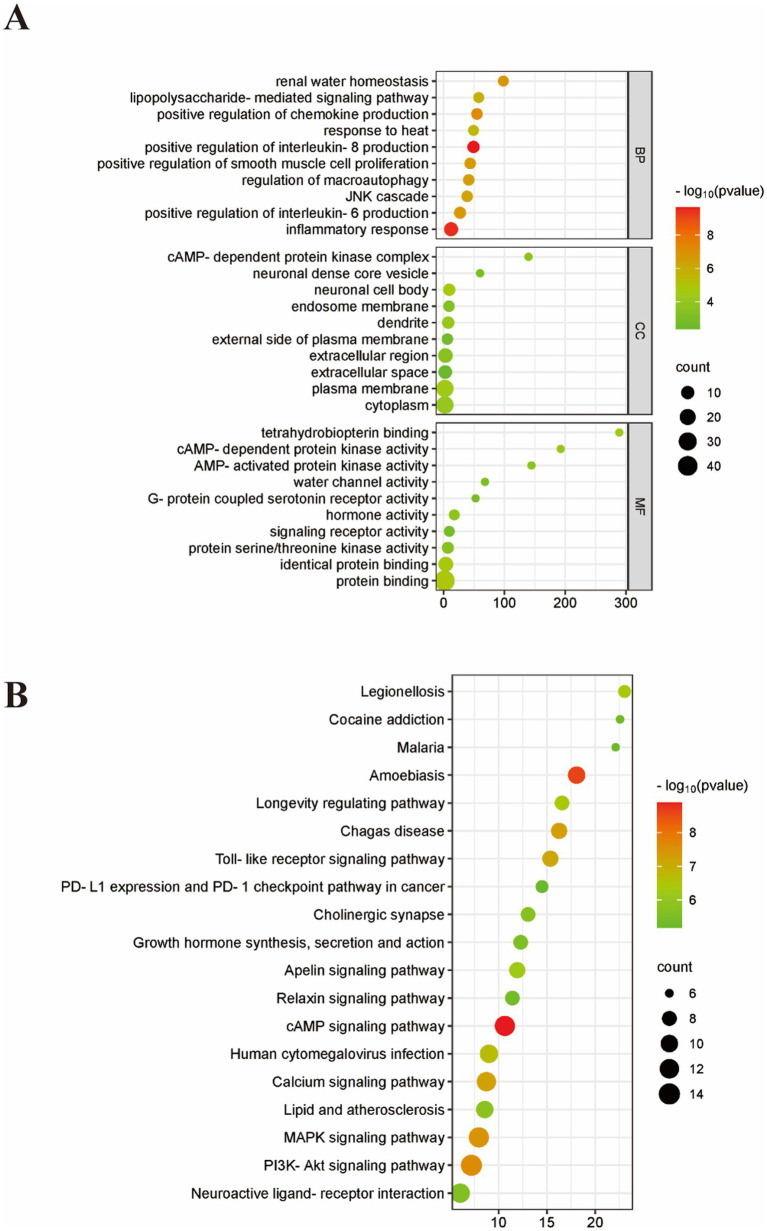
GO and KEGG analysis; **(A)** BP, MF, and CC categories in GO analysis of 49 intersection target. **(B)** Top 20 signaling pathways in KEGG enrichment analysis of 49 intersection target. BP, Biological process; CC, Cellular component; MF, Molecular function.

In the PPI network analysis, the 49 potential targets were subjected to the STRING platform, where only interactions with a high confidence score (exceeding 0.900) were selected (Figure 4). Any isolated nodes were excluded, and the remaining targets were visualized using Cytoscape 3.10.2. The resulting PPI relationship network, comprising 48 nodes and 64 edges, is presented in Figure 5. Targets with the highest degree values, ranking in the top 7, are centrally positioned and consist of NFKB1, IL6, MyD88, TLR4, TNF, TLR2 and IL1B. These nodes exert considerable influence within the PPI network, acting as hubs that connect other targets and are thus identified as core nodes.

### 4.4 GO functional enrichment and KEGG pathway enrichment analysis

In an effort to predict the potential mechanisms by which core acupoint prescriptions may alleviate constipation, a thorough GO functional enrichment analysis was conducted on 49 key targets using the DAVID database. This analysis led to the identification of 257 terms associated with Biological Processes (BP), 37 terms related to Cellular Components (CC), and 41 terms linked to Molecular Functions (MF), culminating in a comprehensive dataset of 347 distinct terms as shown in Figure 5A. The GO functional enrichment analysis yielded significant findings across the three main categories: In the Biological Process (BP) category, the intersected target proteins were predominantly involved in key regulatory processes, including “positive regulation of interleukin-8 production,” “inflammatory response,” “positive regulation of chemokine production,” “renal water homeostasis,” and “positive regulation of interleukin-6 production.”; The Cellular Component (CC) category highlighted the localization of these proteins, with a focus on “neuronal cell body,” “plasma membrane,” “dendrite,” “cytoplasm,” and components of the “cAMP-dependent protein kinase complex.”; Within the Molecular Function (MF) category, the analysis revealed the primary functions of these proteins, such as “protein binding,” “identical protein binding,” “tetrahydrobiopterin binding,” “cAMP-dependent protein kinase activity,” and “hormone activity.”

The KEGG pathway analysis yielded 154 results, and the visualization of the 20 most significant signaling pathways has been featured in Figure 5B. The pathways identified as being of particular importance include the cAMP signaling pathway, amoebiasis, the PI3K-Akt signaling pathway, the MAPK signaling pathway, and pathways associated with Chagas disease.

## 5 Discussion

FC is a complex and refractory intestinal disorder that involves a multitude of factors and mechanisms. While acupuncture has been proven to be a safe and reliable treatment for FC, the full extent of its therapeutic mechanisms remains to be fully understood. Research indicates that acupuncture may alleviate FC through multiple pathways and mechanisms. These include enhancing intestinal motility, regulating the gut microbiota, modulating the brain-gut axis, reducing intestinal inflammatory responses, and alleviating rectal hypersensitivity (15). This approach is characterized by its broad regulatory scope, potent concentrated effects, and minimal adverse reactions. EA is an innovative therapeutic approach that supplements traditional acupuncture with external electrical impulses, thereby amplifying the treatment’s impact. This technique, which merges the principles of acupuncture with electrical stimulation, is gaining prominence for its enhanced therapeutic potential in the field of Traditional Chinese Medicine. EA amplifies traditional acupuncture by introducing electrical stimulation, which is believed to invigorate the flow of qi and blood, stimulate gastrointestinal motility, and facilitate bowel movements (16). Therefore, EA holds positive significance in the treatment of gastrointestinal diseases and is widely used in clinical practice.

In this study, the acupoints that were selected with the highest frequency for EA treatment of FC were Tianshu (ST25), Fujie (SP14), Shangjuxu (ST37), and Zusanli (ST36). We performed an association analysis on 41 acupoints from a dataset comprising 141 acupuncture formulas. The analysis revealed that the combinations of Tianshu (ST25) with Fujie (SP14) and Tianshu (ST25) with Zusanli (ST36) exhibited stronger associations. The third-order association analysis indicates that the combination of Tianshu (ST25), Shangjuxu (ST37), and Zusanli (ST36) exhibits a higher degree of association. Digestive system pathologies typically manifest in the gastrointestinal tract. Tianshu (ST25), situated adjacent to the navel, is a pivotal acupoint for the regulation of Yin and Yang, and is crucial in the treatment of digestive diseases. As the acupoint through which the energy of the Foot Yangming Stomach Meridian flows, it shares a profound connection with the Foot Taiyin Spleen Meridian, reflecting a mutual exterior-interior relationship that facilitates the communication of Qi between the two (17). Known for its efficacy in “regulating the spleen and stomach and harmonizing the visceral energy,” Tianshu is predominantly utilized in the treatment of gastrointestinal conditions, with its application being most prevalent in cases of constipation. The Fujie (SP14) acupoint originates from the “Classic of Acupuncture and Moxibustion” and is located on the Spleen Meridian of Foot-Taiyin. As a point where abdominal Qi tends to gather and form knots, Fujie (SP14) is primarily used for treating conditions related to the aggregation within the abdomen. It can facilitate the flow of Qi through the viscera and regulate the smooth functioning of the body’s energy mechanisms. Stimulating the Fujie acupoint through needling can have a direct effect on the colon located beneath the peritoneum. This local stimulation enhances intestinal motility, speeds up the excretion of fecal matter within the intestines, and thus effectively addresses constipation (18). Shangjuxu (ST37) is considered the lower sea point of the large intestine, adhering to the principle that “sea points treat the internal viscera.” Therefore, Shangjuxu (ST37) acupoint can be used to treat conditions related to the large lntestine (19). EA stimulation at the Shangjuxu (ST37) acupoint is capable of improving intestinal motility dysfunction and can partially restore the function of enteric neurons. The Enteric Nervous System (ENS) contributes to the modulation of intestinal motility by influencing inhibitory neurons (20). Zusanli (ST36), one of the acupoints on the Foot Yangming Stomach Meridian, is also known as the lower sea point and the convergence point of the five shu points. It is believed to have the effects of warming the middle burner, dispersing cold, tonifying the middle and augmenting Qi, strengthening spleen yang, enhancing overall health, promoting the circulation of Qi, guiding Qi downward, and boosting the body’s resistance. Current research indicates that acupuncture at the Zusanli acupoint (ST36) can modulate the bioelectric activity within the gastrointestinal tract, thereby enhancing its motility. This intervention has been shown to alleviate the hypersensitivity of the stomach and intestines and to augment overall gastrointestinal motility. These effects highlight the acupoint’s specific therapeutic role, suggesting a targeted influence on gastrointestinal function (21, 22). Utilizing the Apriori algorithm for the association analysis of acupoints, it is evident that Tianshu (ST25), Fujie (SP14), Shangjuxu (ST37), and Zusanli (ST36) are closely linked, forming the fundamental components of an acupuncture prescription for the treatment of FC.

EA parameters encompass the qualitative and quantitative numerical descriptions of the frequency, intensity, waveform, and stimulation duration of the output pulses emitted by an EA device (14). These parameters are crucial factors that influence the efficacy of acupuncture treatment and modify the mechanisms through which the therapeutic effects are realized. Our research reveals that, upon analyzing the frequency of waveform usage in EA treatments for FC, the dilatational wave emerged as the predominant choice. Studies indicate that dilatational waveforms closely emulate the bioelectrical signals of the human body. By inducing rhythmical muscle contractions, these waveforms are capable of stimulating tissue metabolism and enhancing tissue nourishment (23, 24). Furthermore, the variable nature of dilatational waves is such that they are not readily accommodated by the body. This characteristic helps to maintain an effective stimulus and enhances treatment outcomes, making them advantageous for managing a range of chronic conditions (25).

The frequency of current is a crucial parameter in EA stimulation, significantly influencing the therapeutic effects. Among the literature on EA treatment, 30 publications failed to provide specific parameters for the frequency of stimulation. The standard categorization for EA current frequencies divides them into low frequency (2 Hz to 5 Hz), medium frequency (15 Hz to 30 Hz), and high frequency (50 Hz to 100 Hz). Our research indicates that in the treatment of FC with EA, the selection of current frequency spans from 2 Hz to 100 Hz, with a marked preference for the lower end of the spectrum, particularly favoring 2/15 Hz. Research indicates that low-frequency EA stimulation can enhance bowel motility in constipation by improving colonic myoelectrical activity, reducing the immunoactivity of VIP (Vasoactive Intestinal Peptide), and enhancing the immunoactivity of SP (Substance P), thereby improving constipation (26). EA applied to the ST36 acupoint using dilatational waveforms at low frequencies has been shown to enhance gastrointestinal motility in rats following colonic anastomosis, as well as to alleviate postoperative intestinal inflammation and pain (22). The frequency setting in EA is a crucial parameter, as it modulates the treatment’s impact through distinct neurochemical pathways within the central nervous system (27, 28). Varying frequencies are known to engage different integrative processes via central neural circuits. Additionally, each frequency can trigger the release of specific neurochemicals, leading to a diverse array of physiological effects on the body. Currently, most clinical studies pay insufficient attention to the selection of EA frequencies. The application of these frequencies is often arbitrary, leading to a lack of consistency in the use of EA frequencies in both clinical research and practice. The optimal current frequency for EA in treating FC remains an area that requires further exploration. Building on existing research, future studies could conduct a more in-depth analysis of the efficacy of a 2/15 Hz current frequency for FC treatment.

The findings from the literature analysis reveal that the EA treatment duration for FC predominantly refers to the continuous stimulation time of EA, not including the pre-treatment preparation or post-treatment effect sustenance periods. The predominant continuous stimulation duration is 30 min, representing 90.78% of the cases. Other reported durations also typically fall within the 20 to 30-min range, aligning well with the typical clinical treatment durations currently practiced. In the selection of EA stimulation intensity, due to differences in the EA devices and models used, and the parameters for measuring stimulation intensity being in various units such as current voltage, most of the included literature sets the limit based on the patient’s tolerance. This means that the physician gradually increases the stimulation intensity while asking the patient about their comfort. Once the patient reports that the intensity is too high and unbearable, the intensity is slightly reduced to reach a tolerable level. It is controlled by the patient’s subjective perception of intensity. In clinical practice, due to individual differences among patients and varying levels of sensitivity, there is a wide range of objective values, making it difficult to achieve a clear quantification of stimulation intensity.

Utilizing network acupuncture methods, this research conducted an analysis of the mechanisms underlying the treatment of constipation by the identified core acupoint formulas. Through the construction and analysis of both the “core acupoint prescription-target-constipation” network and the PPI network, key targets were ascertained that are likely pivotal in the biological processes involved in the therapeutic intervention of constipation through core acupoint prescriptions. Nuclear Factor kappa B (NF-κB) is a protein complex that plays a pivotal regulatory role within the cell, particularly in controlling the cell’s response to various stimuli such as stress, cell damage, ultraviolet radiation, and infections from various pathogens (29, 30). Typically, NF-κB is sequestered in an inactive complex with its inhibitor, IκB, existing as either a p60 or p65 subunit. This complex is crucial in modulating inflammatory responses (31). Upon exposure to endotoxins like lipopolysaccharide or pathogen challenge, NF-κB becomes activated by dissociating from IκB, subsequently driving the transcription of pro-inflammatory cytokines, including tumor necrosis factor-alpha (TNF-*α*), interleukin-6 (IL-6), and IL-1β (32, 33). These actions can result in the impairment of both intestinal architecture and function, potentially triggering or intensifying the symptoms of FC. MyD88 (Myeloid differentiation primary response 88) is a protein that plays a key role in the immune system. It is an important adaptor protein in the signaling pathways of Toll-like receptors (TLRs) and the IL-1 receptor family, and it is crucially involved in intracellular signal transduction (34). AKT1, a member of the serine/threonine kinase family, plays a regulatory role in cellular processes such as growth and apoptosis (35).

Inflammatory mediators are recognized as pivotal players in the etiology of FC, with evidence suggesting that they may exert a key influence on both the development and the trajectory of the disease (36). These factors, which include cytokines, chemokines, and other inflammatory substances, can modulate the contractile and relaxant behavior of the intestinal smooth muscle, directly impacting gut motility (37, 38). Moreover, inflammatory mediators can compromise the structural integrity of the intestinal mucosa, diminishing the efficacy of the mucosal barrier and increasing intestinal permeability, which may propagate further inflammatory processes and adversely affect gastrointestinal function (39). The vagus nerve pathway, a key component of the parasympathetic nervous system, plays a crucial role in regulating inflammation and gut motility by releasing acetylcholine that acts on *α*7 nicotinic acetylcholine receptors (α7nAChR) on macrophages, inhibiting the release of pro-inflammatory cytokines and promoting an anti-inflammatory environment (40, 41). The vagus nerve also facilitates communication between the gut and the brain, influencing gastrointestinal function and overall gut health (42, 43). By influencing the vagus nerve, EA may modulate the activity of the enteric nervous system and influence the brain-gut axis, thereby contributing to its therapeutic effects on FC.

Interstitial cells of Cajal (ICC) are pacemaker cells that regulate gut motility and are essential for the propagation of slow waves that coordinate smooth muscle contractions (44). The impairment of ICC has been associated with gastrointestinal motility disorders such as FC. In the context of inflammation, the overexpression of inflammatory factors like TNF-α, IL-1β, IL-6, and nitric oxide (NO) in affected tissues is believed to contribute to the dysfunction of ICC (45). The gut microbiota, which comprises trillions of microorganisms residing in the gastrointestinal tract, plays a significant role in modulating immune responses and gut health (46). Alterations in gut microbiota composition have been associated with FC, as specific microbial taxa can influence intestinal transit time and inflammatory responses. The gut microbiota can also produce metabolites that affect the gut-brain axis, further influencing motility and inflammation (47). EA may exert its effects through these pathways by modulating the microbiota and influencing ICC function, which in turn can impact gastrointestinal motility and contribute to the treatment of FC.

An integrated analysis of the GO and KEGG enrichment outcomes indicates that the core acupoint prescription influence on constipation predominantly intersects with several key signaling pathways, including the cAMP signaling pathway, the PI3K-Akt signaling pathway, the MAPK signaling pathway, the Calcium signaling pathway and The Toll-like receptor signaling pathway. Cyclic adenosine monophosphate (cAMP) plays a role in mediating a multitude of biological processes (48). An elevation in intracellular cAMP levels can lead to the activation of cAMP-dependent protein kinase A (PKA), which in turn modulates metabolic activities. The phosphoinositide 3-kinase (PI3K) to protein kinase B (Akt) signaling cascade participates in a diverse array of biological processes and is intricately linked to cellular inflammation and the process of oncogenesis (49). Mitogen-Activated Protein Kinases (MAPKs) constitute a family of serine/threonine-specific protein kinases integral to cellular processes such as proliferation, differentiation, apoptosis, and inflammatory responses (50). They form a significant inflammatory signaling cascade. Research indicates a profound connection between the MAPK pathway and conditions of inflammation and cancer (51). Activated by inflammatory mediators, the MAPK signaling pathway serves as an extracellular signal transduction mechanism that modulates the synthesis of inflammatory cytokines (52). In summary, the above evidence suggests that the therapeutic effects of acupuncture treatment are due to the effective combination of acupoints, which exert a multi-target and multi-pathway, contributes to the amelioration of symptoms associated with FC.

## 6 Limitations

In this study, the number of included literature sources was limited. Nonetheless, the acupoint spectrum for EA treatment of constipation, derived through data mining, aligns with clinical practice and adheres to the fundamental theories of Traditional Chinese Medicine (TCM) acupuncture. However, the literature suffers from unclear concepts regarding EA stimulus dosage, lacks uniform quantification standards, and provides an insufficient basis for quantification. Future research should aim to standardize the stimulus parameters of EA devices and establish appropriate parameter standards for related conditions. This will ensure the optimal combination of parameters for clinical treatment, making them applicable for clinical practice and enhancing clinical efficacy.

## 7 Conclusion

This study applies data mining to identify patterns in acupoint selection and EA parameters for treating FC. It highlights a core acupoint formula including Tianshu (ST25), Fujie (SP14), Shangjuxu (ST37), and Zusanli (ST36), and recommends the EA parameter set of a dilatational waveform at 2/15 Hz for 30 min. The research indicates that this approach may influence multiple targets and pathways, such as NFKB1, IL6, TNF, MYD88, TLR4, AKT1, IL1B, and various signaling pathways, offering a comprehensive strategy for constipation treatment. The findings provide a foundation for optimizing EA clinical protocols and inspire further studies on EA’s therapeutic mechanisms on constipation.

In summary, the study offers insights and a theoretical framework for clinical and basic research on EA treatment for FC.

## Data Availability

The original contributions presented in the study are included in the article/supplementary material. Further inquiries can be directed to the corresponding authors.

## Footnotes

^1^ http://www.uniprot.org/

^2^ https://www.genecards.org/

^3^ http://db.idrblab.net/ttd/

^4^ https://www.disgenet.org/

^5^ http://ehbio.com/test/venn/#/

^6^ https://david.ncifcrf.gov/

^7^ http://www.bioinformatics.com.cn/
